# Origins location of the outflow tract ventricular arrhythmias exhibiting qrS pattern or QS pattern with a notch on the descending limb in lead V1

**DOI:** 10.1186/s12872-017-0561-y

**Published:** 2017-05-15

**Authors:** Cong Lin, Cheng Zheng, De-Pu Zhou, Xiao-Wei Li, Shu-Jie Wu, Jia-Feng Lin

**Affiliations:** Department of Cardiology, Second Affiliated Hospital of Wenzhou Medical university, 109 Xueyuan Road, Wenzhou, Zhejiang 325000 China

**Keywords:** Premature ventricular contraction, Radiofrequency catheter ablation, Electrocardiogram, Ventricular outflow tract

## Abstract

**Background:**

Ventricular outflow tract(VOT) ventricular arrhythmias(VAs) presenting qrS pattern or QS pattern with a notch on the descending limb in lead V1 were consistently thought of arising from the commissure between left and right coronary cusp (L-RCC) by previous studies. However, we found they could originate from other anatomic structures in VOT. This study aimed to investigate the exact origin of this kind VAs.

**Methods:**

Forty-nine patients of VOT premature ventricular contrations/ventricular tachycardia(PVCs/VT) with lead V1 presenting qrS pattern or QS pattern with a notch on the descending limb undergoing successful radiofrequency catheter ablation(RFCA) in our center were analyzed.

**Results:**

12-lead electrocardiogram(ECG) of these PVCs/VT were summarized. Among these PVCs/VT, 37 cases exhibited qrS morphology in lead V1, 12 cases presented QS pattern with a notch on the descending limb in the same lead. Based on the successful ablation sites, these PVCs/VT were divided into 2 groups: (1)Right ventricular outflow tract(RVOT) group (26 cases), and (2) Left ventricular outflow tract (LVOT) group(23 cases, 4 cases originating from the left coronary cusp(LCC), 2 from the right coronary cusp(RCC), 16 from the L-RCC, 1 from the area inferior to LCC(ILCC)). The ECG characteristics of each PVCs/VT were analyzed. Among these PVCs/VT, applying the precordial transitional zone index(TZ index) < 0 to predict LVOT origin was demonstrated with sensitivity of 95.65%, specificity of 96.15%, positive predicting value(PPV) of 95.65% and negative predicting value(NPV) of 96.15%. In LVOT group, further applying the r, R, m,or Rs morphology in lead I to predict L-RCC and RCC origin was demonstrated with sensitivity of 94.44%, specificity of 60.00%, PPV of 89.47% and NPV of 75.00%.

**Conclusions:**

Ventricular outflow tract PVCs/VT with lead V1 presenting qrS pattern or QS pattern with a notch on descending limb not only arising from L-RCC, but also RVOT, LCC, RCC and ILCC. Combining TZ index and QRS morphology in lead I to predict origin site of these kind VAs is a convenient, simple and reliable method and facilitates the RFCA procedure.

## Background

Idiopathic ventricular arrhythmias(VAs), including premature ventricular contractions(PVCs) and ventricular tachycardia (VT), are the most common arrhythmias found in patients with normal heart structure. The most common origin of PVCs/VT is the right ventricular outflow tract (RVOT), followed by left ventricular outflow tract (LVOT), atrioventricular annulus (tricuspid and mitral valve annulus), left ventricular septum, and the main pulmonary trunk. Notably, radiofrequency catheter ablation(RFCA) has been demonstrated as a safe and effective approach in treating PVCs/VT from multiple origins [1–9]. However, the catheter ablation of PVCs/VT arising from the left and right coronary sinus cusp commissure(L-RCC) has been rarely reported [10, 11]. In 2008, Japanese scholars Yamada et al. [11] revealed that a qrS pattern in leads V1–V3 suggests origin sites of VAs at the L-RCC . However, in our clinical practice, we found that PVCs which had qrS pattern or QS pattern with a descending limb notching in lead V1 did not all originate from L-RCC, some of them had other ventricular outflow tract (VOT) origins, such as left coronary cusp (LCC), right coronary cusp (RCC) or RVOT. In the present study, we aimed to investigate the exact origins of VOT PVCs who have qrS pattern or QS pattern with a descending limb notching in lead V1.

## Methods

From January 2008 to March 2016, radiofrequency catheter ablation was performed on 855 patients (mean age, 48.15 ± 17.21 years) with symptomatic VOT PVCs/VT at the second affiliated hospital of Wenzhou Medical University. Before RFCA, all patients underwent conventional biochemical tests, chest X-ray, echocardiography, and treadmill test. When needed, the right, left ventricle and/or coronary angiography (CAG) as well as cardiac magnetic resonance was performed to exclude structural heart disease. Patients who had one of the following were excluded from our study: (1) Serious dysfunction of heart, lung, liver and/or kidney and coagulation disorders that could not tolerate surgery; (2) A history of viral myocarditis or myocardial infarction within 6 months; (3) A history of stroke within 6 months; (4) Malignant tumor; (5) Severe thoracic deformity; (6)Being elderly (> 85 years). Before ablation, all patients had discontinued the anti-arrhythmic therapy for more than 5 half-lives. All patients signed the informed consent form and agreed to the electrophysiological examination and radiofrequency ablation treatment. All these treatments patients received were considered standard care for their conditions.

### Electrocardiographic analysis

All PVCs/VT allowed analysis of the QRS complex morphology, independent of the P and T waves. During clinical arrhythmias, the following ECG characteristics were focused on: 1) QRS axis; 2) QRS morphology in precordial leads; 3) QRS morphology in lead I; 4) Precordial transition zone; 5) Precordial transition zone index [12](refers to the PVCs/VT precordial transitional zone minus the sinus rhythm precordial transitional zone); 6) R wave duration index, R/S amplitude index in lead V2 [13] (The R wave duration index is calculated by dividing the longer R-wave duration in lead V1 or V2 by the QRS complex duration, the R/S amplitude ratio in leads V1 and V2 is calculated using the amplitude of the QRS complex peak to the isoelectric line ratio the amplitude of the QRS nadir to the isoelectric line, the R/S amplitude index refers to the greater value of R/S wave amplitude ratio in lead V1 or V2, in this study, for all PVCs/VT presented qrS pattern or QS pattern in lead V1, which resulted in a small R wave or non R wave in lead V1, we calculated R wave duration index, R/S amplitude index in lead V2); 7) QRS duration.

### Electrophysiology study

A 6-F quadripolar catheter was delivered to the right atrium from the femoral vein and then placed across the atrioventricular valve to map the largest His potential. Activation mapping and pace mapping were performed in both right ventricle and left ventricle to locate the origin of VAs with the application of 7F, 4-mm-tip ablation catheters. The method of activation mapping and pace mapping have been described in detail in our previous work [1, 4–8]. When clinical PVCs/VT did not appear spontaneously at the beginning of the electrophysiology study, burst pacing from right ventricular apex (set at basic cycle lengths of 600, 500, 430 ms respectively) with the intravenous isoproterenol infusion(2–4 μg/min) was initiated to induce the arrhythmias.

### Radiofrequency catheter ablation

The target site for ablation was defined as sites mapped with the earliest ventricular activation and exhibiting good pace match (≥11/12 lead match). Once a target site was located, RFCA application was attempted. When a target site was mapped near the aortic root, angiography of the aortic sinus cusps and coronary artery was performed before ablation to investigate the distance from the tip of ablation catheter to those structures. The energy of ablation should not be released within 5 mm of the ostium of coronary artery. When the temperature-controlled catheters were utilized, radiofrequency applications were delivered with a target temperature of 55–60 °C and maximum power output of 30–40 W. If the impedance produced by temperature-controlled catheters was too high, the irrigated-tip catheters with a 17 ml/min saline flow rate were utilized instead, with the goal to achieve a target temperature of 43 °C and maximum power output of 30–35 W. If the VT or PVCs accelerated or decelerated during the first 10s of energy releasing on the target sites, the radiofrequency delivery was lasted for 60 to 180 s. If not, the radiofrequency delivery was ceased, and another target site was searched for. When a target site was mapped with a His potential or located in an area within 5 mm from the site recording the biggest His potential, radiofrequency energy was quitted.

Programmed electrical stimulation and intravenous isoproterenol were both performed before withdrawal all catheters and sheaths to reassure the complete elimination of PVCs/VT.

During the whole procedure, the activated clotting time was maintained between 250 and 300 s by intravenous administration of heparin.

### Procedure Success and Follow-Up

We define ablation success was defined as (1) absence of spontaneous or provoked VAs at end of procedure, and (2) absence of pre-procedure VAs on 48-h ECG monitoring postablation off antiarrhythmic drugs. Transthoracic echocardiogram and 24-h holter were performed immediately before discharge and 3 and 6 months after ablation in each patient.

### Statistical analysis

Continuous data were manifested as mean ± standard deviation (±*s*) and were compared using analysis of variance (ANOVA) and the student’s t-test, while categorical data are expressed as number and percentage, and were compared using the X^2^-test or Fisher method. *P* < 0.05 indicated a significant difference.

## Result

### Population Characteristics

Among 855 VOT PVCs/VT, 49 (4.68%) cases presented a qrS pattern or QS pattern with a notch on the descending limb in lead V1. Among the 49 VOT PVCs/VT, 22 patients were male and 27 were female, average aged 50.42 ± 15.56 (23 ~ 74) years, had disease duration of 29.89 ± 28.67 (3 ~ 132) months, 18 patients (36.73%) had a history of hypertension, 6 (12.24%) had diabetes mellitus, 1 (2.04%) had coronary heart disease with coronary stent implanted. All these patients had taken at least one anti-arrhythmic drugs, including metoprolol, propafenone, mexiletine and amiodarone, with an average number of anti-arrhythmic drugs 1.8 ± 0.5. 24 h holter of these patients were recorded at least once before RFCA and showed the average number of PVCs 22,451 ± 8931 (10,113 ~ 46,872). Echocardiography showed average left ventricular ejection fraction (LVEF) of 65.67 ± 3.70 (50 ~ 70)%, and average left ventricular end diastolic diameter (LVEDd) of 48.88 ± 2.97 (45 ~ 59) mm.

### RFCA results

**Fig. 1:**
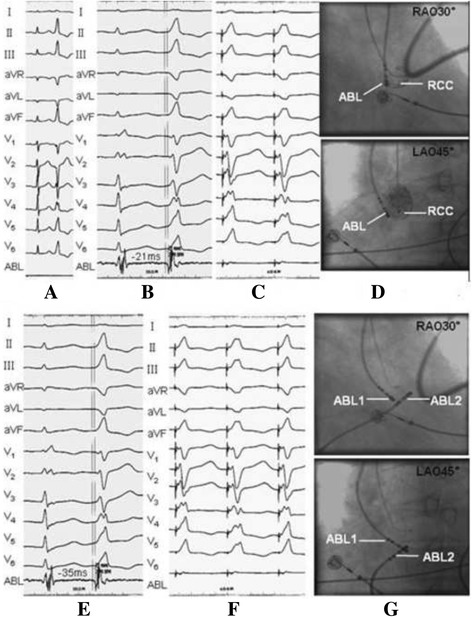
Ablation of PVCs with lead V1 presenting QS pattern with notching in descending limb. ECG showed frequent PVCs, in which the QRS complex showed a complete left bundle branch block (CLBBB) pattern with an inferior axis, r pattern in lead I, QS pattern with notching descending limb in lead V1-V2, R pattern in leads II, III, aVF and V4 ~ V6, QS pattern in leads aVR and aVL, the precordial transition zone between V3 and V4 (**a**). According to the characteristics of QRS complex in V1 ~ V2 combined with Yamada’s report, the PVCs was firstly considered arising from L-RCC. When activation mapping performed in ASCs, we mapped an relatively earlier ventricular potential preceding the spontaneous PVCs QRS complex onset by 21 ms (**b**) in RCC, and pacing this site showed a perfect pace match(**c**). X-ray image showed the ablation catheter in RCC(**d**). X-ray image showed another ablation catheter was located in RVOT and corresponding to the tip of ablation catheter in RCC(**g**). We then mapped a ventricular potential preceding the PVCs QRS onset by 35 ms in RVOT (**e**), pacing the potential result in a perfect QRS match to spontaneous PVCs(**f**). Current discharging on this site for approximately 3 s completely abolished PVCs. Note: RCC = right coronary cusp

The forty-nine patients of ventricular outflow tract (VOT) PVCs/VT with lead V1 presenting qrS pattern or QS pattern with a notch on the descending limb were all successfully eliminated by RFCA in our center, 26 cases were ablated in RVOT, the other 23 cases were ablated in LVOT (4 from LCC, 2 from RCC, 16 from L-RCC, and 1 from ILCC), no procedure-related complications occurred. In addition, all 49 patients were free of arrhythmias without anti-arrhythmic drugs during the following up of 12 ± 3 months. RFCA procedure of a VOT PVC presenting QS pattern with a notch on the descending limb in lead V1 was showed in Fig. 1.

### ECG characteristics

**Fig. 2:**
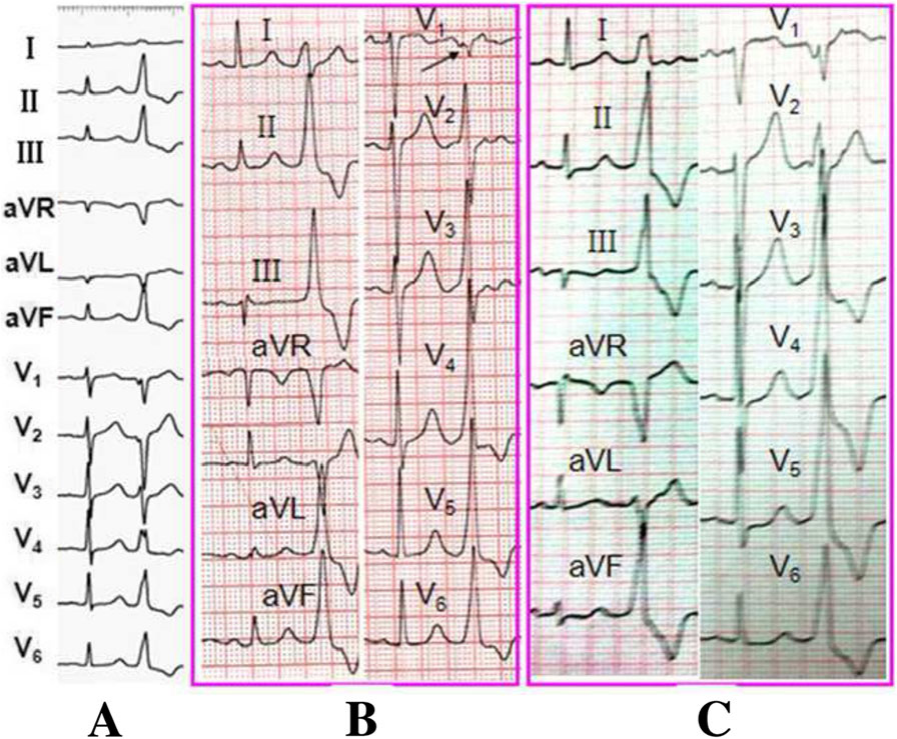
**a**. Transition index of the precordial leads is 3.5–2.5 = 1 (greater than 0), lead I had the positive wave of m -pattern, indicating the origin of of RVOT; **b**. Transition index of the precordial leads is 2–3.5 = −1.5 (less than 0), V2 had R wave with a duration ratio > 50%, and an amplitude ratio > 30%, indicating the ASC origin, lead I had the Rs pattern, eventually it was identified as having the L-RCC origin; **c**. Transition index of the precordial leads is 2.5–3.5 = −1 (less than 0), V2 had a R wave with a duration ratio > 50% and an amplitude ratio > 30%, combined with lead I which showed R pattern positive wave, eventually it was identified as having the RCC origin

In 49 cases, all PVCs/VT showed inferior axis, monophasic R pattern in leads II, III and aVF, QS pattern in leads aVR and aVL, in lead V1, 37cases had qrS pattern, the other 12 cases had QS pattern with a notch on the descending limb; in lead V2, 9 cases had qrS or qRS, 4 had QS with a notch in the descending limb, and 36 had rS or RS. The detail ECG characteristics of each patient are shown in Table 1. Typical ECGs of PVCs/VT originating from RVOT, L-RCC and RCC were showed in Fig. 2.

**Table 1:** QRS complex morphology of PVCs

NO	Origin	QRS axis	QRS complex morphology						Transition zone	V2 R wave Duration index > 50% R/S amplitude ratio > 30%	Transition index*	QRS duration (ms)
			V_1_	V_2_	V_3_	V_4_	V_5_ ~ V_6_	I				
1	RVOT	down	qrS	qrS	rS	R	R	m	V_3_ ~ V_4_	no	1	130
2	LCC	down	qs	RS	Rs	R	R	Rs	V_2_	yes	−1.5	130
3	L-RCC	down	qrS	rS	RS	Rs	R	Rs	V_3_	yes	−0.5	120
4	RVOT	down	qrS	rS	rS	R	R	m	V_4_ ~ V_5_	no	0.5	140
5	RVOT	down	QS	QS	rS	R	R	m	V_4_ ~ V_5_	no	1	135
6	RVOT	down	qrS	rS	rS	R	R	rs	V_3_ ~ V_4_	no	1	140
7	RVOT	down	qrS	rS	rS	R	R	m	V_3_ ~ V_4_	no	0.5	140
8	RCC	nornal	qrS	R	R	R	R	R	V_1_ ~ V_2_	yes	−1.5	122
9	LCC	down	QS	Rs	R	R	R	rs	V_1_ ~ V_2_	yes	−1	142
10	RVOT	down	qrS	rS	rS	RS	R	rs	V_4_	no	0.5	140
11	RVOT	down	qrS	rS	Rs	R	R	rs	V_2_ ~ V_3_	yes	0.5	126
12	RVOT	down	qrS	rS	rS	RS	R	rs	V_4_	no	1	146
13	L-RCC	down	qrS	rS	rS	Rs	R	rsr’	V_3_ ~ V_4_	no	−0.5	138
14	RVOT	down	qrS	rS	rS	RS	R	rs	V_3_ ~ V_4_	no	0.5	142
15	RVOT	down	qrS	rS	rS	RS	R	rs	V_3_ ~ V_4_	no	1	140
16	LCC	down	QS	rS	rS	Rs	R	rs	V_3_ ~ V_4_	no	−0.5	136
17	L-RCC	down	QS	rS	RS	R	R	m	V_3_	yes	1	142
18	L-RCC	down	qrS	RS	R	R	R	R	V_2_	yes	−1	140
19	RVOT	down	qrS	rS	RS	R	R	m	V_3_	no	−0.5	140
20	L-RCC	down	qrS	RS	R	R	R	m	V_2_	yes	−1	142
21	L- RCC	down	qrS	RS	R	R	R	m	V_2_	yes	−0.5	136
22	RVOT	down	qrS	qrS	RS	R	R	m	V_3_	no	0	132
23	RVOT	down	qrS	rS	Rs	R	R	m	V_2_ ~ V_3_	yes	0	129
24	RVOT	down	QS	qrS	rS	Rs	R	rs	V_3_ ~ V_4_	no	0.5	132
25	L- RCC	down	QS	RS	R	R	R	R	V_2_ ~ V_3_	yes	−1	138
26	RVOT	down	qrS	rS	RS	R	R	m	V_3_	no	0.5	150
27	RVOT	down	qrS	rS	RS	R	R	R	V_3_	no	0	136
28	RVOT	down	qrS	rS	RS	R	R	m	V_3_	no	0	142
29	ILCC	down	qrS	Rs	R	R	R	Rs	<V_1_	yes	−2	128
30	RVOT	down	qrS	rS	rS	R	R	qr	V_3_ ~ V_4_	no	1	132
31	L- RCC	down	qrS	rS	R	R	R	R	V_2_ ~ V_3_	yes	−1	147
32	RVOT	down	qrS	rS	rS	Rs	R	rS	V_3_ ~ V_4_	no	0	142
33	L- RCC	down	qrS	RS	R	R	R	R	V_2_	yes	−1	138
34	L- RCC	down	qRS	qRS	R	R	R	m	<V_1_	yes	−2	136
35	RVOT	down	qRS	rS	Rs	R	R	R	V_2_ ~ V_3_	yes	0.5	156
36	RVOT	down	qrS	qrS	rS	Rs	R	m	V_3_ ~ V_4_	no	0.5	142
37	L- RCC	down	qrS	qRS	R	R	R	R	V_2_ ~ V_3_	no	−0.5	132
38	LCC	down	QS	RS	Rs	R	R	rs	V_2_	yes	−1.5	133
39	L- RCC	down	qrS	RS	R	R	R	R	V_2_	yes	−1	130
40	L-RCC	down	qrS	RS	R	R	R	R	V_2_ ~ V_3_	yes	−0.5	152
41	RVOT	down	QS	rS	rS	R	R	R	V_3_ ~ V_4_	no	0.5	142
42	RVOT	down	qrS	rS	rS	R	R	R	V_3_ ~ V_4_	no	0.5	119
43	L-RCC	down	qrS	RS	R	R	R	R	V_2_ ~ V_3_	yes	−0.5	130
44	RCC	down	qrS	qrS	RS	R	R	R	V_2_ ~ V_3_	no	−0.5	120
45	L-RCC	down	qrS	RS	R	R	R	R	V_2_	yes	−1	138
46	RVOT	down	QS	qrS	rS	rS	R	rs	V_4_ ~ V_5_	no	1.5	136
47	RVOT	down	QS	QS	Rs	R	R	r	V_2_ ~ V_3_	no	0	132
48	RVOT	down	QS	QS	RS	R	R	r	V_2_ ~ V_3_	no	0.5	134
49	L-RCC	down	qrS	qrS	R	R	R	R	V_2_ ~ V_3_	no	−0.5	130

Annotation:*L-RCC* Left coronary cusp-Right coronary, *ILCC* Inferior of left coronary cusp,* Transition index = PVC transition-sinus transition.

### Differential diagnostic value of specific ECG characteristics in identifying the origin of outflow tract PVCs/VT having qrS pattern or QS pattern with notching on the descending limb in V1

**Fig. 3:**
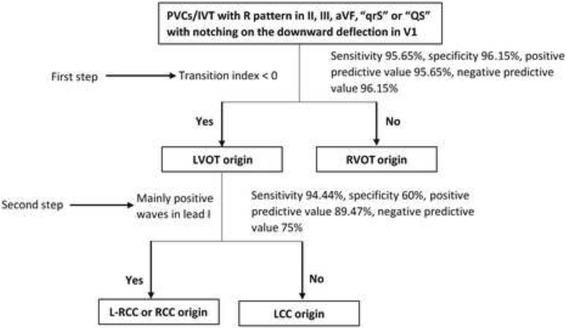
Flow chat of identification of origins of outflow tract PVCs/VT having “qrS” pattern or “QS” pattern with a notch on the descending limb in lead V1

Among these 49 VOT PVCs/VT exhibiting qrS pattern or QS pattern with a notch on the descending limb in lead V1, in RVOT group, 10 VAs presented precordial transitional zone ≤ V3, while, in LVOT group, 20 VAs did, applying precordial transitional zone ≤ V3 to predict LVOT origin showed a sensitivity of 86.96%, specificity of 61.54%, positive predicting value(PPV) of 66.67% and negative predicting value(NPV) of 84.21%. In RVOT group, 3 VAs exhibited R wave duration index > 50% and R/S amplitude index > 30% in lead V2; in LVOT group, 18 VAs exhibited, using R wave duration index > 50% and R/S amplitude index > 30% in lead V2 to predict LVOT origin revealed a sensitivity of 78.26%, specificity of 88.46%, PPV of 85.71%, and NPV of 82.14%. In RVOT group, only 1 PVCs presented precordial transitional zone index(TZ index) < 0, while in LVOT group, 22 PVCs did, applying the precordial TZ index < 0 to predict LVOT origin was demonstrated with sensitivity of 95.65%, specificity of 96.15%, positive predicting value(PPV) of 95.65% and negative predicting value(NPV) of 96.15%. Among the 23 LVOT PVCs/VT exhibiting qrS pattern or QS pattern with a notch on the descending limb in lead V1, we found 2/2 RCC and 15/16 L-RCC VAs present m, r, R or Rs morphology in lead I, further applying the m, r, R or Rs morphology in lead I to predict L-RCC and RCC origin was demonstrated with sensitivity of 94.44%, specificity of 60%, PPV of 89.47% and NPV of 75.00%, Tables 2 and 3. Based on the ECG characteristics and ablation results of these PVCs/VT, a flow chat for identification of origins of VOT PVCs/VT having “qrS” pattern or “QS” pattern with a notch on the descending limb in lead V1 was proposed by our center, Fig. 3.

**Table 2:** Summaries of electrocardiogram characteristics of the different origins

Origin	Number	QRS pattern in leads V1		Transition zone		leads V2 R wave Duration index > 50% and R/S amplitude ratio > 30%		Transition index		QRS pattern in leads I	
		qrS	QS	≤V3	>V3	yes	no	<0	≥0	R、r、m or Rs	rs or qr
RVOT	26	20	6	10	16	3	23	1	25	16	10
LVOT	23	17	6	20^a^	3	18	5	22 ^a^	1	19	4
LCC	4	/	4	3	1	3	1	4	/	1	3
L-RCC	16	14	2	15	1	13	3	15	1	15	1
RCC	2	2	/	1	1	1	1	2	/	2	/
ILCC	1	0/1	/	1	/	1	/	1	/	1	/

Annotation: compared with RVOT, ^a^ *p* < .001.

**Table 3:** Differential diagnostic value of standard ECG in identifying the origin of outflow tract PVCs having qrS or QS pattern with notching on the downward deflection in V1 (*n*, %)

ECG indicators	Sensitivity	Specificity	Positive predictive value	Negative predictive value
Precordial lead transitional zone index <0, LVOT origin [12]	22/23(95.65)	25/26(96.15)	22/23(95.65)	25/26(96.15)
Precordial lead transitional zone ≤ V_3,_ LVOT origin	20/23(86.96)	16/26(61.54)	20/28(66.67)	16/19(84.21)
Lead V2 R wave Duration index > 50% R/S amplitude index > 30%, LVOT origin	18/23(78.26)	23/26(88.46)	18/21(85.71)	23/28(82.14)
r, m,R or Rs wave in lead I, LCC-RCC or RCC origin	17/18(94.44)	3/5(60.00)	17/19(89.47)	3/4(75.00)

## Discussion

In the present study, we found that VOT PVCs/VT exhibiting qrS morphology or QS morphology with a notch on the descending limb in lead V1 could arise from RVOT, LCC, RCC, L-RCC, ILCC respectively, and be effectively treated with RFCA. Combining TZ index and QRS morphology in lead I to predict origin site of these kind VAs is a convenient, simple and reliable method and facilitates the RFCA procedure.

Several previous studies showed that VOT PVCs/VT exhibiting qrS morphology or QS morphology with a notch on the descending limb in lead V1 rose from L-RCC. Yamada et al. [10] performed successful ablation of 146 cases of PVCs with a ventricular outflow tract origin. Among these 146 cases, 5 cases had qrS pattern in lead V1 ~ V3, all of which were successfully ablated from L-RCC; the rest 141 cases arose from other sites of outflow and none of them have qrS pattern in V1 ~ V3. In addition, Yamada et al. paced multiple sites in the aortic root in the control subjects demonstrated that only pacing from the L-RCC could reproduce a qrS pattern in leads V1–V3. Thus, they concluded that the ventricular arrhythmias with qrS in the right chest leads had a high specificity in discriminating the L-RCC origin with RVOT and other LVOT origins. Bala et al. [11] investigate the electrocardiographic characteristics related to ventricular arrhythmias arising from the L-RCC. Among 35 consecutive patients with ventricular arrhythmias arising from the aortic cusp region, they found a QS morphology in lead V1 with a notch on the descending limb was exhibited in 15 of 19 ventricular arrhythmias arising from the L-RCC compared to 2 of 18 ventricular arrhythmias from other aortic cusp sites, thus they proposed that common features of ventricular arrhythmia originating from L-RCC was QS pattern with a notch on the descending limb in lead V1, and the precordial transitional zone was prior to lead V3. Both studies indicated VOT PVCs with V1 presenting qrS or QS pattern with notching on the descending limb mainly originating from the L-RCC.

In the past 8 years we had performed successfully RFCA in 49 PVCs/VT presenting qrS or QS pattern with notching on the descending limb in lead V1. We confirmed that 26 PVC cases had RVOT origin, and 23 had LVOT origin (16 from L-RCC, 4 from LCC, 2 from RCC and 1 from ILCC, respectively). According to the ECG diagnosis criteria proposed by Bala et al. [10], the majority cases in our study were supposed to have a L-RCC origin, however, only 16 cases actually arose from L-RCC origin. However, we found combining TZ index and QRS morphology in lead I, the origin of VOT PVCs with qrS or QS pattern with notching on the descending limb in lead V1 can be precisely predicted.

We used to think that PVCs/VT with a LVOT origin has an early TZ before lead V3, however, recent studies demonstrated that the predicting value of TZ ≤ V3 for LVOT was actually not so good owing to the complex anatomical structure of VOT and cardiac rotation. Ouyang et al. [12] reported that for VOT PVCs/VT, the morphology of QRS complexes in leads V1 ~ V2 may be used to identify RVOT or LVOT origin, they suggested that the PVCs were of LVOT origin when the R/S amplitude index >0.3 or R wave duration index >0.5, otherwise it was from RVOT. Yoshida et al. [14] once proposed that utilizing the V2S/V3R index to distinguish left VOT-VAs from right VOT-VA, when the V2S/V3R index ≤1.5, a LVOT origin was considered. Another study by Yoshida N et al. [13] found that precordial TZ index (refers to the PVCs/VT precordial transitional zone minus the sinus rhythm precordial transitional zone) < 0 had a higher sensitivity, specificity and accuracy in predicting LVOT-originated ventricular arrhythmias. Liu Z et al. [15] suggested that multiple intercostal recordings resulted in the interpretation of the depolarization vector of the outflow tract ventricular arrhythmia in a more comprehensive way, and proposed the combined TZ index ≤0.25 helped identify the ASC origin more accurately. In our study, we referred to the ECG criteria proposed by ouyang et al. and Yoshida et al. for they were simple and convenient, the predictive ability of precordial TZ < 3, precordial TZ index < 0, and R wave duration index > 50% and R/S amplitude index > 30% for LVOT origin was compared with each others, the TZ index < 0 was demonstrated to be the best ECG criterion among them. In the purpose of rapidly and accurately locating VOT PVCs with lead V1 presenting qrS pattern or QS pattern with a notch on the descending limb before RFCA, we suggested the precordial TZ index < 0 should be primarily adopted to differentiate the LVOT origin from the RVOT origin. In addition, in our study, we found that Ouyang et al.’ criterion R wave duration index >0.5 and R/S amplitude index > 0.3 did not show a good ability for identifying LVOT origin, thus we suggested that Ouyang criterion which mainly based on QRS morphology in lead V1-V2 was actually not suitable for this special kind PVCs/VT.

The QRS morphology in lead I was further adopted to differentiate PVCs origin within ASCs. Ouyang et al. [12] showed that ventricular arrhythmias with different origins within the aortic sinus cusps(ASCs) exhibited certain differences in lead I, PVCs from the LCC region typically have an rS in lead I, whereas, PVCs from the RCC typically are positive and notched in lead I. In our study, we found the ventricular arrhythmias with LCC origin in lead I had mainly negative waves, while the ventricular arrhythmias with RCC and L-RCC origin in lead I had primarily positive waves with r, R, m or Rs pattern. The distinctive morphology in lead I of these ASC ventricular arrhythmias was mainly caused by the their depolarization direction, depolarization of the ventricular arrhythmias with LCC origin was usually toward the opposed direction of lead I, thus resulted in a mainly negative wave, while the depolarization direction of the ventricular arrhythmias with L-RCC or RCC origin was usually toward the same direction of lead I, thus resulted a mainly positive waves. Therefore, the morphology of the QRS complex in lead I may help to further determine the origin of ventricular arrhythmias exhibiting qrS or QS pattern with notching in the descending limb in lead V1. When the r, m, R or Rs morphology in lead I was applied to predict L-RCC and RCC origin in our study, it was demonstrated with sensitivity of 94.44%, specificity of 60%, positive predictive value of 89.47% and negative predictive value of 75.00%.

Based on our observation, we concluded that VOT PVCs/VT with V1 presenting qrS patten or QS pattern with a notch in descending limb did not all originate from L-RCC, when predicting the exact origins of this kind PVCs/VT, both the TZ index and morphology in lead I should be taken into account.

Limitations of this study included, ① location of PVC/VT origins were mainly based on X-ray images (aortic sinus angiography) and three-dimensional mapping system, nonetheless, the advanced intracardiac ultrasound technique was not applied; ② our sample size was small, which could lead a certain level of sampling bias. Thus, a future study with a large cohort is warranted to corroborate our findings.

## Conclusion

In the current study, we found that combining TZ index < 0 and QRS morphology in lead I can precisely predict origin site of VOT VAs exhibited qrS morphology or QS morphology with a notch on the descending limb in lead V1.

## Abbreviation

ANOVA: Analysis of variance
ASCs: Aortic sinus cusps
CAG: Coronary angiography
CLBBB: Complete left bundle branch block
ECG: Electrocardiogram
ILCC: Inferior to left coronary cusp
LCC: Left coronary cusp
L-RCC: Commissure between left and right coronary cusps
LVEDd: Left ventricular end diastolic diameter
LVEF: Left ventricular ejection fraction
LVOT: Left ventricular outflow tract
NPV: Negative predicting value
PPV: Positive predicting value
PVCs: Premature ventricular contrations
RCC: Right coronary cusp
RFCA: Radiofrequency catheter ablation
RVOT: Right ventricular outflow tract
TZ: Transitional zone
VOT: Ventricular outflow tract
VT: Ventricular tachycardia
